# Narrative Review of Novel Nicotine-Delivery Systems and Emerging Pharmaceuticals for Tobacco Cessation

**DOI:** 10.3390/medicines13020019

**Published:** 2026-06-12

**Authors:** Srilekha Mutukula, Taylor Gagne-Hatfield, Zachary R. Dunbar

**Affiliations:** Lake Erie College of Osteopathic Medicine at Elmira, 1 LECOM Pl, Elmira, NY 14901, USA

**Keywords:** cessation, e-cigarettes, ENDS, snus, varenicline, bupropion, cytisine, population health, tobacco control

## Abstract

**Background**: Debate continues to swirl around the effectiveness of novel nicotine-delivery products such as snus and e-cigarettes as tobacco cessation aids. The purpose of this review is to quantify the state of research on modern products, including e-products, and established or developing pharmaceuticals on assisting nicotine users in achieving cessation. **Methods:** This study relied on a comprehensive assessment of research articles, clinical trials, drug approvals, and textbook material available via PubMed, Ovid Wolters Kluwer, and Wiley. We utilized Python 3.14.2, Anaconda3, the ShinyWeb App, and Py.Litstudy to investigate the selected literature. Our key study elements are product evolution and cessation behavior associated with e-cigarettes, snus, nicotine gum, nicotine dermal patches, bupropion, varenicline, and cytisine. **Results:** This manuscript assessed 144 manuscripts published between 1952 and 2025. E-cigarettes and snus, while containing some limited cessation benefit, were not identified to be effective enough at attaining cessation (when used exclusively) to be prescribed as cessation tools. Cytisine was identified as having very similar cessation outcomes to established pharmaceuticals such as varenicline. **Conclusions:** Since their iteration, e-cigarettes and snus products were marketed as cessation aids. This review found that there is scant evidence to support that modern snus and e-cigarette products work as cessation aids when used in exclusion of other more traditional approaches to cessative aid. Additionally, more modern pharmaceuticals such as cytisine may have benefit as solo cessation tools over novel nicotine-delivery products.

## 1. Introduction

Evidence of the causal relationship between tobacco use and increased risk for lung cancer has existed for over sixty years [1], and in the intervening decades, ongoing use of nicotine via combustible and non-combustible products has been associated with a plethora of other cancerous processes besides lung (such as breast [2], ovarian [3], and cervical [4] cancers) as well as other comorbidities such as elevated cholesterol, blood pressure, and increased risk of heart disease [5]. Further, use of any substance containing nicotine (i.e., combustible cigarettes, electronic products, hookah, cigars, cigarillos, heat-not-burn products, or smokeless products) is associated with a greater risk of perpetuating economic disparity, as previous studies have detected that excess income being dedicated to tobacco increased economic burden on nicotine users in Latin America, Canada, Nigeria, China, India, and Japan [6,7,8,9,10,11]. The disparate economic burden combined with increasing risk for oncologic and pulmonic/cardiac comorbidities provides a potent motivator for many global tobacco and nicotine users to quit using their products of choice [12,13,14,15]. In response to both shrinking usage of combustible products globally in favor of novel nicotine-delivery systems [16] and the widespread marketing of emerging products as conventional tobacco cessation aids [17], the modern tobacco regulatory and prescriptive framework for physicians and legislators is muddled.

Nicotine cessation is the process of quitting the use of products containing nicotine, including traditional cigarettes, cigars, cigarillos, smokeless tobacco, and electronic nicotine-delivery systems (ENDSs). Achieving tobacco cessation is a critical public health goal, given the high rates of preventable morbidity, mortality, and economic costs associated with nicotine addiction [18]. Tobacco use contributes significantly to chronic diseases such as cardiovascular disease, cancer, and respiratory illness, and its burden on public health is especially pronounced in both in low- and middle-income countries and among youth populations globally [19,20]. Understanding the evolving market for nicotine and tobacco consumption is therefore critical to achieving an ability to prevent future tobacco use and assist in the cessation of continued nicotine dependence.

The use of traditional combustible tobacco products such as cigarettes, waterpipe (hookah), and cigars has decreased over the past twenty years globally due to comprehensive and intensive international tobacco control efforts [21]. However, with the preponderance of increasingly smaller battery units and other improvements in electronics over the same period, emerging products such as electronic nicotine-delivery systems have supplanted cigarette use in some countries [22]. With the arrival of these products came targeted, focused messaging aimed at promoting their use as cessation aids [23], a finding that was closely associated with JUUL brand e-cigarettes [24]. However, achieving complete nicotine cessation through e-cigarette use has not been confirmed as a primary modality for abstention from tobacco [25,26], as will be discussed in greater detail below. Further, the onset of e-cigarettes, snus, and other products allowed tobacco companies to re-enter advertising spaces with messaging intended for young adults, which had a profound impact on increasing nicotine use in these populations [27,28,29,30]. Adolescents are an especially vulnerable population as early nicotine exposure increases the likelihood of long-term nicotine dependence and complicates cessation in adulthood [19]. Neuro-developmentally, it disrupts prefrontal cortex development, steepens the trajectory toward addiction, and increases cognitive impairment and psychiatric risk [31]. To address this, a range of evidence-based cessation strategies has been established. First-line options include over-the-counter nicotine replacement therapies such as transdermal patches and nicotine gum, and prescription medications like bupropion and varenicline [32]. In parallel, non-pharmacological approaches such as group support, motivational interviewing, and cognitive behavioral therapy have been widely studied and implemented [33].

Digital platforms and social media are increasingly being leveraged for behavior change, especially in youth populations [34]. Yet, these same platforms have also fueled adolescent uptake through targeted pro-vape advertising, as seen in the controversies surrounding Juul [24]. The recent 2025 U.S. Food and Drug Administration “JUUL ruling,” which reversed a 2022 ban to allow certain products back on the market after determining adult benefits outweigh youth risks, underscores this tension [35]. Digital platforms, therefore, may both contribute to increasing ENDS use while also posing as a potential means to reduce use. Contingency management approaches, where digital rewards are given in response to verified abstinence, may be particularly effective for adolescents, whose decision-making is highly influenced by short-term rewards due to developmental differences in prefrontal cortical control [19,36]. These gamified systems remain underutilized in standard clinical care but represent an important innovation in adolescent cessation strategies.

Globally, cessation approaches are becoming more personalized, blending pharmacologic tools with behavioral reinforcement tailored to an individual’s demographic background and preference [37]. Yet, despite this progress, major challenges persist, particularly in communities with limited resources. Many regions continue to face critical gaps in provider training, limited availability of cessation medications, and underdeveloped public health infrastructure [8]. These limitations are especially evident among smokeless tobacco users and individuals using newer tobacco alternatives like electronic nicotine-delivery systems, where evidence-based cessation protocols are less well-established [20]. This divide regarding resource availability will underscore future prevention efforts, which will need to address specific, individualized community needs. The aim of this assessment is to construct a narrative framework of the many challenges facing the modern tobacco “quitter”; specifically, the glut of available products that may or may not have peer-reviewed evidence in support of their use as cessation aids.

## 2. Methodology

### 2.1. Literature Review

This manuscript comprises a literature review of PubMed, Ovid, Wolters Kluwer, and Wiley. The intent of our search was to establish a chronology of evolutionary products that contain nicotine as well as conventional therapeutics to aid in cessation. All references were held in and generated by Zotero 7.0.29 for Mac (Digital Scholar, Vienna, VA, USA). By constructing this narrative assessment, the underlying aim is to evaluate and compile a wide review of the literature on cessation and prevention published in the English language. Selected manuscripts were limited to those written in English, published and hosted on one of the aforementioned sites, and addressing only cessation, use rationale, and product pharmacology. The subcategories provided herein shall define the evolution and use of these products.

Our primary goal was to establish a comprehensive narrative of the emergence of novel tobacco products; to promote a reasonable study population we limited our scope to electronic nicotine-delivery systems and snus products, as these are the most prominent forms of tobacco used among modern youth around the globe [38,39]. Our aim was to synergize the history, social drivers, and factors associated with the prevalence of the use of these products with the underlying pharmacological tools available for physicians and public health professionals to intercede against ongoing nicotine use—namely, transdermal patches, gum, bupropion, varenicline, and emerging products such as cytisine, semaglutide, psilocybin, tricyclic antidepressants, essential oils, and even e-cigarettes themselves. The temporal scope of our assessment was practically limited to within the past 10 years, with several exceptions for seminal papers of their era, such as the Cutler and Loveland associative study of 1954 [1] and the developmental papers on bupropion in the mid-1980s to late 1990s [40,41,42]. Our search parameters are defined in detail below:

### 2.2. Search

**Figure 1:**
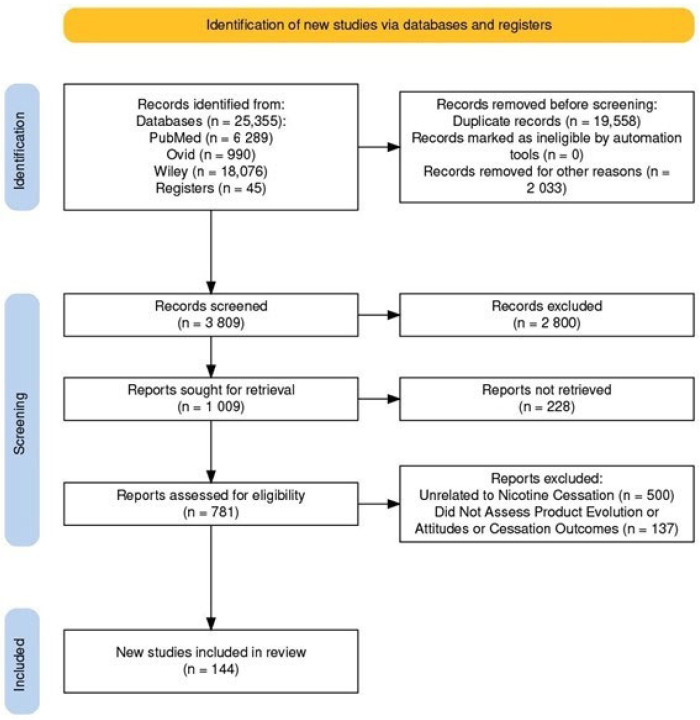
PRISMA flow diagram for document selection.

We structured our assessment on a PRISMA-like model utilizing Python 3.14.4. We applied the following Boolean terms into PubMed, Ovid, Wolters Kluwer, and Wiley and received the total number of documents outlined in Table 1:

As listed above, our total querying approach resulted in 25,400 ‘hits’, from which we underwent a PRISMA-style approach to reducing the number of selected works to a more accessible number. Our selection criteria included: English Language, Accessible Full Text via our Institution (including books), and topical relevance defined as pertaining to the history of the product and its applicability to nicotine or tobacco cessation. In practice, this included review articles such as ours, published clinical trial data, randomized control trials, meta-analyses, and relevant book excerpts. We utilized the Shiny WebApp Development site to generate the following PRISMA diagram [43] and the full account of our selection is depicted in Figure 1 below:

Our initial query arrived at the unworkable total of 25,400 documents, of which 45 were clinical trials. Using Python 3.14.2, we were able to deduplicate 19,558 entries, and 2033 records were removed as they were references to physical texts that our library did not have access to. Of the remaining 3809 records, 2800 were not in English, and were therefore excluded rather than over-relying on translation software. In the cohort of 1009 remaining documents, 228 reports were not retrieved due to the full text being unattainable. The remaining 781 were assessed for relevance (defined as related to Nicotine or Tobacco cessation, product evolution, attitudes towards use, or direct cessation outcomes) resulting in the remaining 144 studies included in our assessment. We utilized Anaconda 3, Py.litstudy, and Jupyter Notebook (Long Beach, CA, USA, https://pypi.org/project/litstudy/) (Accessed 20 January 2026) to visualize the publication year, author list, primary topic, and to formulate word associative clouds with our cohort data, as shown below.

## 3. Results and Discussion

### 3.1. Literature Characteristics

**Figure 2:**
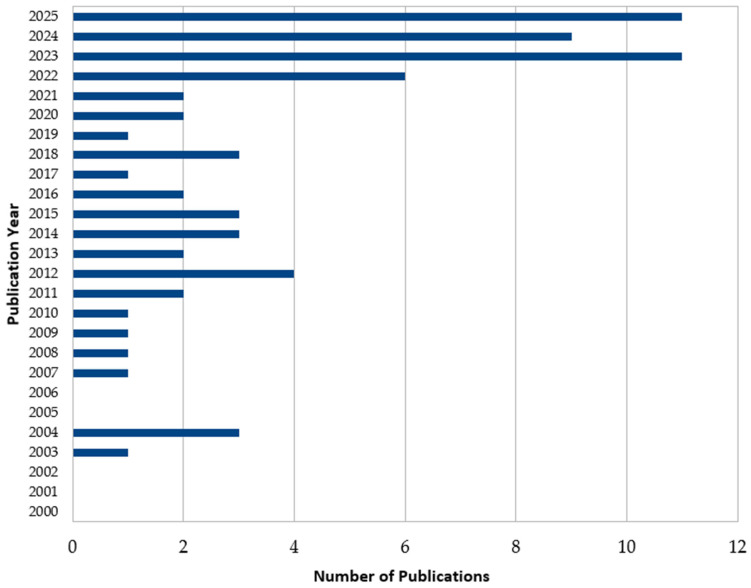
Number of publications per year (excluding publication date earlier than 2000).

Excluding the seven studies in our inclusion criteria that predated the year 2000, most of the studies we assessed were observed to take place between the years 2022–2025, as shown in Figure 2:

This finding may suggest recency bias in publication until it is also considered that emerging products like cytisine, e-cigarettes, and snus products did not gain widespread use until the mid-2010s, resulting in a lag time in peer-reviewed publications on those products. 

**Figure 3:**
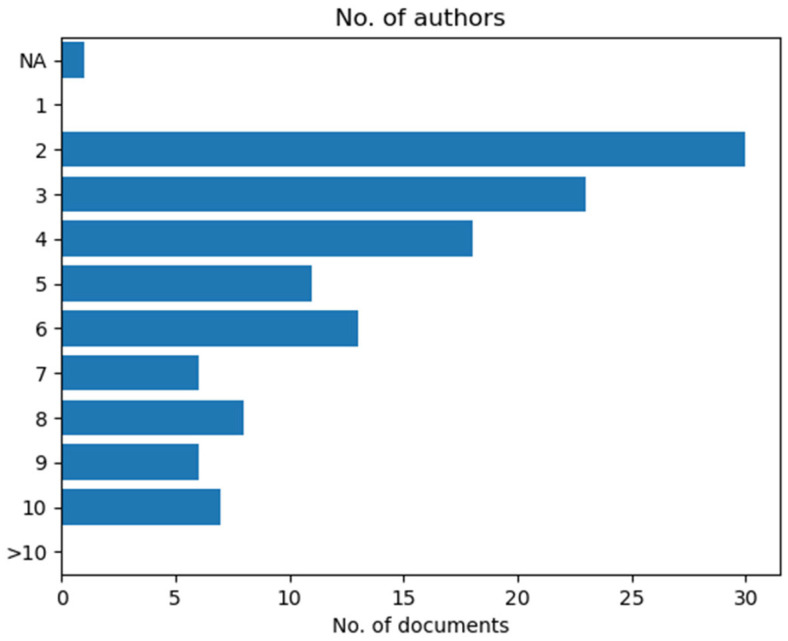
Histogram: Number of authors per publication.

Figure 3 above shows the number of authors per manuscript evaluated. The two documents that did not have any sole authors available were both U.S. Food and Drug Administration Drug Approval sheets for Chantix and Wellbutrin, respectively, that form most of the data series described in below. The remaining author lists have the highest distribution at two authors per paper before attenuating up to as many as ten authors per listing. As the frequency of assessed documents with greater than three co-contributors rises, so does the likelihood that there are multiple institutions involved with a given assessment of tobacco or nicotine cessation using ENDS, snus, patches, gum, or the pharmaceuticals described below.

**Figure 4:**
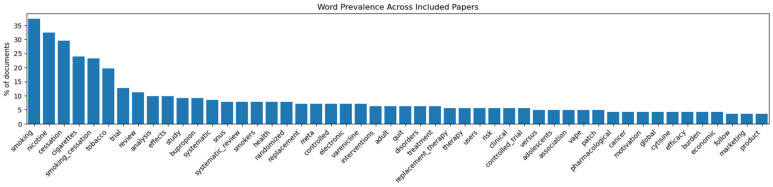
Word prevalence across included papers.

**Figure 5:**
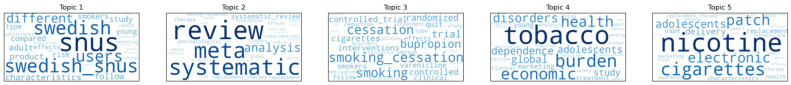
Word cloud of popular word associations by topic.

Figure 4 and Figure 5 on the next page both highlight the assessment of topics across all of the papers that were surveyed in this assessment. Py.Litstudy allows users to scrape full manuscripts of each paper included in an assay to create a topic word cloud of the most common terms used across each paper, and we utilized that strategy to generate the figures on the following page.

The word cloud in Figure 5 corroborates the trend seen in Figure 4 nicely. The four most common topics across all the surveyed data include product types: “Electronic, Patch, Snus, Bupropion”, populations: “Smokers, Adolescents, Users,” and study design elements: “Review, Systematic, Trial.” These groupings by themselves may hint at how researchers speak about products (i.e., prevalent use of the term “users”) compared to how those that engage with nicotine products view themselves. An interesting future avenue for study would be to assess potential stigma facing smokers and vapers when they engage with scientific media. Stigma may color respondents’ answers regarding use frequency, potentially introducing variance in some of the studies that form the backbone for the discussion in the following sections.

### 3.2. Novel Nicotine-Delivery Systems

#### 3.2.1. Electronic Nicotine-Delivery Systems

Electronic nicotine-delivery products have undergone a series of evolutionary steps following their initial entry into the market; however, the primary elements of the electronic cigarette have not changed over time. All ENDS products contain, at their simplest construction, a vessel for “e-liquid” containing nicotine solution, a battery, and a vaporizing mouthpiece [44]. The earliest products, the first generation, looked very similar to conventional tobacco cigarettes and were designed to be disposable [45]. Second and third generation products introduced the vaporizing chamber, which was either pre-filled (in second generation) or refillable (in the third) [46]. These eventually gave way to the modern fourth-generation ENDS products, which place the coil element in a sealed pre-filled vaporizing chamber containing either free or salt-concentrate nicotine, a proprietary mixture of propylene glycol and vegetable glycerin, and depending on market, flavoring ingredients resulting in either a particular flavor or ‘unflavored’ (menthol or ‘tobacco’) taste [47]. Even with reduced flavor availability, modern generational ENDSs had already massively dominated the nicotine market [29,48], leading to continued discussion and debate on the health and safety of these emergent devices.

Electronic nicotine products first entered the cessation space in the second and third generations, where the refillable chamber was advertised both as a cessation aid [49,50] and a means to experiment with use [50]. In the early era of electronic cigarettes, the lax regulatory environment and targeted messaging to adolescents and young adults were responsible for a large increase in ENDS use [24,30]. Users in the mid-2010s would flock to social media to perform recorded ‘vape tricks’ where they would inhale and exhale vapor on camera, usually to in sync with music [51,52]. Cottage industries sprung up around custom flavorings, including ‘do-it-yourself’ mixtures that appealed to a much wider audience than conventional tobacco [53]. These unique admixtures led to toxicological profiles that varied widely and side effects from home-use that were unpredictable, such as alcohol poisoning from nicotine and alcohol mixtures [54]. At the time, there were few regulations in place on the flavoring of e-liquids, meaning that the industry was incentivized to incorporate flavorings into their marketing strategy. A key reason that individuals cited wishing to vape was because they had access to flavorings with ENDS products that they did not have with tobacco products [55]. Similarly, This an increasing number of new users reported interest in experimentation with ENDSs for the novelty, the taste, because they perceived that it was safer than smoking conventional products, and because they could use these devices in places that they were not allowed to smoke cigarettes [55,56]. As mentioned above, ENDS use rose precipitously during this era of product evolution and exploration, leading to a new generation of users experiencing nicotine dependence.

Perhaps the most pre-eminent discussion in public health spaces around ENDS products relates to the debate surrounding the efficacy of ENDSs as cessation aids. As discussed above, this debate stems from tobacco industry marketing and targeted messaging, obfuscating some of the ongoing risks of continued use. Indirect exposures to heavy metals [57,58] and other potentially hazardous ingredients have fallen to the background of discussion of use of these devices as a means to quit conventional tobacco. In our literature review, we uncovered ten different randomized control trials that assessed the likelihood of cessation from sole ENDS use or ENDS use in combination with replacement therapies or other therapeutics, and found the following associations:

The data presented above suggest that from a prescription perspective, the sole use of ENDS products as a cessation aid had mixed results. Each of the studies mentioned above were randomized control trials in a variety of global populations with an equally variant underlying frequency of tobacco use. Interestingly, there was one additional study in favor of ENDS use as a cessation tool; Xu et al. observed in their 2023 randomized control trial an Odds Ratio for continued nicotine use of 0.73 (95% CI: 0.68, 0.77), or, that ENDS use was longitudinally protective against ongoing nicotine use; however, that study was funded in part by JUUL Labs Inc, shedding some concern over the reproducibility of the findings. That said, while some evidence does exist that ENDS products can be helpful to some conventional tobacco users, the risk of poly-use or continued ENDS use in place of abstinence has also been observed to occur in meaningful rates [25,26,37]. It is also worth noting here that the research compiled in Table 2 is localized to North America, Europe, and Oceana, and there is a need for greater research in Asian and Southern Hemisphere nations on ENDSs’ applicability to smoking cessation in those communities. The relatively small (<1500 participant) sample sizes above also highlight the need for more longitudinal randomized large-sample assessments of ENDSs’ applicability as a standalone cessation tool. These trade-offs are enough to encourage public health officials and medical providers to continue to seek informed community-based care for attaining cessation.

#### 3.2.2. Snus Products

Snus products (also known as “Swedish snus”) originated in the early 19th century and were made from ground tobacco, water, salt, and potash [68]. Its convenience, especially for people doing manual labor, along with its low cost, helped it gain popularity. Unlike other forms of smokeless tobacco, snus is placed under the upper lip, which reduces saliva production and eliminates the need for spitting. When health warnings about smoking began to circulate in the 1960s, the use of snus in Sweden started to rise again [69]. While it was never officially marketed as a cessation aid, many users reported switching from cigarettes to snus, and evidence has since shown it may aid in cessation, at least in Scandinavian populations [70,71]. In the 1970s, growing concerns about product quality and new food-grade regulations led manufacturers to modernize production, resulting in stricter hygiene practices, ingredient controls, chemical testing, and eventually the establishment of the GothiaTek standard [72] that continues to define quality and safety benchmarks today.

Swedish snus delivers nicotine efficiently through the lining of the mouth, with bioavailability ranging from about 40% to 60%, depending on the brand; for example, products like General, Catch Mini, and Catch Dry Mini offer around 40% bioavailability, while Catch Licorice reaches closer to 60%, likely due to more effective absorption [73]. Nicotine levels in the bloodstream also vary by product, with General snus producing peak plasma concentrations near 29 ng/mL—comparable to the higher end of what is seen in cigarette smokers. Other brands deliver slightly lower levels but still provide meaningful nicotine exposure. The relatively high pH of snus, typically between 7.8 and 8.5, increases the amount of free-base nicotine, which is absorbed more quickly through the mucous membranes; further, the concentration of un-ionized vs. ionized nicotine varies from market to market [74]. Several snus brands deliver more than twice the nicotine exposure of 2 mg Nicorette gum, which likely explains why snus can provide faster and more sustained relief from cravings [75]. This pharmacokinetic profile may help explain its effectiveness in managing withdrawal symptoms and its appeal to smokers looking for a less harmful alternative. More recently, nicotine pouch products such as Zyn brand—which contain no tobacco leaf but rely on similar oral absorption pathways—have become the dominant player in the U.S. domestic market, reflecting both the popularity of smokeless nicotine-delivery systems and their potential role in tobacco harm reduction [76,77]. As is the case with ENDS products, the popularity of snus in the modern market has led to ongoing debate regarding its role in cessation.

While snus is not risk-free [78], there is evidence that it lacks the respiratory risks and contains fewer carcinogens than conventional burning tobacco [79]. There are reasonable concerns that snus may encourage tobacco use among youth or lead to poly-use [80], but evidence suggests most snus users in Scandinavia are former smokers and overall smoking rates have declined, indicating snus may help some adults transition away from cigarettes [72]. Ethically, providing smokers with accurate information about snus’s lower risks respects their autonomy and could reduce public health harm. However, concerns remain about tobacco industry marketing practices [81] and the potential diversion of resources from established tobacco control efforts. Consequently, snus is not widely promoted by physicians, but many agree it holds promise as a regulated alternative for smokers unable to quit entirely, provided measures are in place to prevent youth uptake and discourage dual-use [79].

Use of snus, like other nicotine products, can lead to physical dependence, leading to withdrawal symptoms such as cravings, restlessness, irritability, and trouble concentrating during the abstinence phase [81]. These findings confirm that snus can trigger a recognizable withdrawal syndrome. Long-term data from Sweden indicate that while some users eventually stop using snus, many continue over long periods; those who reported both physical and psychological benefits after switching were more likely to stick with it, while those who did not perceive such advantages were more likely to stop [82]. Unfortunately, support for those trying to quit snus is limited, with most advice available only online, and little research existing on the long-term effects of quitting snus after daily use. As such, snus presents a complex picture: it holds promise as a harm-reduction tool but also carries its own risk of long-term nicotine dependence.

### 3.3. Nicotine Transdermal Patches

Nicotine transdermal patches were developed in the late 1980s and introduced in the early 1990s as a pharmacologic strategy to support smoking cessation. Researchers developed this patch in response to increasing evidence that nicotine is highly addictive and that safer ways to deliver it were urgently needed, and in 1991, the U.S. Food and Drug Administration approved the first prescription nicotine patch, followed by over-the-counter availability in 1996 [83]. Due to their simplicity, tolerability, and proven efficacy, patches quickly became a cornerstone of smoking cessation efforts.

Nicotine patches deliver the drug transdermally (via the skin), allowing gradual absorption into the systemic circulation while bypassing hepatic first-pass metabolism. This enables steady plasma nicotine levels over 16 to 24 h, depending on the formulation, and a standard 21 mg/day patch reaches a steady-state plasma concentration of approximately 17 ng/mL within 2–4 h and maintains stable levels throughout the day; this avoids the sharp peaks and crashes associated with smoking [84,85]. This slower, more stable absorption profile is considered advantageous for reducing dependence, as it helps prevent the rapid reinforcement cycle typically triggered by cigarette use [86].

Clinicians commonly recommend nicotine patches as a first-line treatment for tobacco cessation because they are effective, easy to use, and generally safe [87]. According to U.S. Public Health Service guidelines, nicotine replacement therapies like patches “approximately double the chances of quitting successfully compared to placebo or no treatment” [88]. The once-daily application and steady nicotine release reduce the potential for misuse, making patches a convenient choice for many patients. Dosage is usually tailored to the individual’s smoking habits, with heavy smokers often starting at 21 mg and gradually tapering over 8 to 12 weeks; for patients experiencing breakthrough cravings, clinicians may recommend combining the patch with faster-acting nicotine products such as gum or lozenges to enhance cessation success [89].

Nicotine patches work by keeping a steady level of nicotine in the body, which helps take the edge off cravings and reduces withdrawal symptoms [90]. People often find that common symptoms like irritability, anxiety, restlessness, and trouble concentrating become more manageable when nicotine levels stay above the threshold needed to ease those effects [91]. While some users may experience mild side effects, like skin irritation where the patch is applied or occasional sleep issues, these effects are usually temporary and much less distressing than the symptoms of quitting cold turkey [92]. As mentioned above, many patch users find titratable nicotine withdrawal relief that does aid cessation.

Most individuals discontinue patch use after completing an 8 to 12-week tapering schedule, which is designed to gradually reduce nicotine dependence and support long-term abstinence [89]. However, individuals with high nicotine dependence or increased relapse risk may benefit from extended use or multiple courses. Adherence is a critical predictor of cessation success, as consistent use is strongly associated with higher quit rates [93]. Combining nicotine patch therapy with behavioral interventions further enhances the likelihood of sustained abstinence [94]. In a randomized trial in New Zealand, Walker et al. (2020) found that participants using nicotine patches in combination with nicotine-containing e-cigarettes experienced modestly higher quit rates compared to those using patches alone or with nicotine-free e-cigarettes [94]. Importantly, the study also observed that some participants continued to use both nicotine patches and e-cigarettes simultaneously, highlighting that dual-use is a common behavior and may need to be addressed in clinical guidance. These findings support the idea that flexible, combination approaches, particularly when paired with behavioral support, may help individuals achieve sustained abstinence while managing cravings and withdrawal more effectively.

### 3.4. Nicotine Gum

Nicotine gum entered the mass-market in the late 1970s as one of the first forms of nicotine replacement therapy; initially designed for use by Swedish naval submariners who could not smoke while submerged, it was soon recognized as a promising cessation tool for the general population [95,96]. Its development marked an important shift in how tobacco use is understood—not just as a habit, but as a physical addiction driven by the pharmacologic effects of nicotine. Nicotine replacement therapies like nicotine gum were designed to temporarily take the place of nicotine from cigarettes, helping to ease withdrawal symptoms and reduce cravings, which makes it easier for people to begin the journey toward quitting completely [90]. Over time, gum became one of several tools available to support quitting, alongside patches, sprays, inhalers, and lozenges, each offering a different modality of nicotine delivery [90]. While patches provide steady systemic nicotine and inhalers mimic hand-to-mouth cues, gum not only delivers nicotine orally but may also relieve the urge to chew, gnash teeth, or engage in other mechanical or oral stimming behaviors that often emerge during withdrawal [97]. This dual pharmacologic and behavioral role likely contributes to gum’s enduring appeal as part of comprehensive cessation programs aimed at reducing dependence and improving long-term success rates.

The mechanism of action of nicotine gum relies on buccal absorption. When chewed, nicotine is released and absorbed through the oral mucosa into the systemic circulation, bypassing hepatic first-pass metabolism; this allows for a controlled and slower rise in plasma nicotine levels compared to cigarette smoking [84]. Typically, nicotine is absorbed over 20 to 30 min, with peak plasma concentrations from a 4 mg gum reaching approximately 10 ng/mL—much lower than the 25–60 ng/mL associated with a single cigarette [84,98]. Although the bioavailability is moderate, the absorption is sufficient to reduce cravings and withdrawal symptoms without triggering the rapid reinforcement cycle that contributes to dependence [88]. Thus, while nicotine from gum is less readily available than inhaled nicotine, its pharmacokinetic profile is therapeutically effective and safer. 

Nicotine gum is widely promoted by physicians as a first-line pharmacological treatment for tobacco dependence; it is considered a simple, cost-effective, and practical tool that enhances and reinforces smoking cessation efforts, with evidence demonstrating significantly higher 12-month abstinence rates compared to no gum-use [88]. The highest success rates are seen when nicotine gum is combined with longer, structured follow-up, highlighting its value in comprehensive clinical cessation programs [99]. Additionally, nicotine gum provides flexible, on-demand dosing that allows patients to manage cravings as they arise, making it particularly beneficial for individuals who require behavioral substitution in tandem with nicotine replacement; this format allows for easy manipulation and individualized use, further supporting its effectiveness and acceptability in real-world settings [90].

Regarding the duration of gum-use, most individuals follow a structured tapering schedule, typically over 8 to 12 weeks; heavier smokers generally start with the 4 mg gum and taper down to the 2 mg dose as cravings lessen [100]. Over several weeks or months, the number of daily doses is reduced until the gum is no longer needed [90]. While some individuals may extend use beyond the standard period, especially those with persistent cravings, continued use of nicotine gum is considered safer than returning to cigarette smoking [101]. Nicotine gum, alongside nicotine patches, are well established and well-researched cessation modalities that, in combination with more direct pharmaceutical involvement if needed, have a storied history of efficacy in reducing nicotine dependence.

### 3.5. Established Pharmaceutical: Bupropion

Bupropion was originally developed in 1974 as an atypical antidepressant under the name amfebutamone by Burroughs Wellcome [102] in response to the adverse effects of common agents at the time, namely monoamine oxidase inhibitors (MAOIs), selective serotonin reuptake inhibitors (SSRIs), and tricyclic antidepressants (TCAs) [103]. When bupropion was approved for use in major depressive disorder in 1989, its mechanism of action was not completely understood, but it was concluded to have neurotransmitter effects different from the commonly used agents of the time [104], particularly regarding dopamine and norepinephrine [40]. It is now understood to inhibit reuptake of dopamine (DA) and norepinephrine (NE) through noncompetitive binding to and resulting antagonism of nicotinic acetylcholine receptors (nAChRs), with some evidence showing specific activity within the nucleus accumbens and locus ceruleus [103]. This antagonism, which primarily involves the M2 ion channel region surrounding the central receptor pore, thus lowering probability of the channel opening, resulting in receptor desensitization, functionally inhibiting nAChR activity [105].

Following oral administration, bupropion is rapidly absorbed from the GI tract, with intestinal absorption reported to be 100%; from here, it is extensively distributed throughout the body via plasma protein binding; however, total bioavailability is estimated to be as low as 5–20% [41]. This is largely attributed to first-pass metabolism, specifically via CYP2B6, which is so extensive that less than 10% of the parent drug is excreted in urine or feces and only 1% is excreted unchanged [41]. While data related to elimination of the different formularies of bupropion has yet to be agreed upon, the most common formulas have an elimination half-life of 18 h.

In 1997, one year after the approval of the sustained-release formulation under the brand Wellbutrin [102], bupropion was approved for use as a smoking cessation aid after clinical trials demonstrated improved abstinence from nicotine, despite similarly reported withdrawal symptoms between the placebo and test groups [42]. Later, bupropion was also shown to significantly delay relapse and reduced weight gain attributed to cessation [106]. Prior to this, nicotine cessation attempts were supplemented with behavioral therapy and nicotine replacement therapies (NRTs), and although there was significant benefit of NRT and behavioral therapy [32], it is established that both bupropion monotherapy and bupropion in conjunction with NRT and behavioral therapy is statistically superior to NRT and behavioral therapy alone [107]. Bupropion’s role in smoking cessation is due, in part, to its effect on dopamine impacting the mesolimbic and corticolimbic pathways, which inhibits the addictive effect of nicotine while also providing craving relief [108]. Furthermore, bupropion has also been shown to noncompetitively inhibit many neuronal nicotinic receptors, resulting in a functional blockade of nicotine’s nicotinic effects [109].

Bupropion represents the first non-nicotine drug used in smoking cessation [109], and since its approval, other agents have been explored. Since bupropion was originally developed as an alternative treatment for major depressive disorder, other antidepressants, such as sertraline [110], nortriptyline, and selegiline [111] have been implemented for use in smoking cessation. However, efficacy of these agents is questionable, since studies focus on smoking cessation in the setting of depressive disorders in which these pharmaceuticals are indicated [111,112,113]. Because of this, SSRIs are not recommended for use in smoking cessation [89,114]. The search for effective pharmacotherapeutics for cessation continued to evolve past bupropion in more recent years, as will be described in the next section.

### 3.6. Established Pharmaceutical: Varenicline

Varenicline, sold under the trade name “Chantix”, was developed based on the partial agonist activity of cytisine, a natural plant alkaloid, at the α4β2 nAChR [115,116]. As an oral agent, varenicline is nearly completely absorbed in the proximal GI tract with a bioavailability around 90%, and undergoes minimal metabolism before it is excreted in urine unchanged with an elimination half-life of about 24 h [117]. Varenicline’s competitive agonism results in submaximal dopamine release in areas where nicotine is active [118], leading to remission of withdrawal symptoms while simultaneously preventing full agonism via consumed nicotine, resulting in dulling of the reinforcing effects of nicotine use [116]. Varenicline also retains its partial antagonism in the presence of full agonist nicotine and mitigates the dopamine-enhancing effects of nicotine [115]. Varenicline is a well-established line of care for nicotine cessation.

In trials, varenicline out-performed bupropion and NRTs as monotherapy when compared to placebo, with 21.8% of patients displaying biochemically verified continuous abstinence at 6 months compared to 16.2% and 15.7% for bupropion and NRTs, respectively [118]. Compared to established combination therapies, varenicline alone has been shown to be at least as effective as combination NRTs, such as a patch plus a short-acting NRT like nicotine gum, showing similar quit rates over a 6-month period.

When it was originally released to the public, there was concern regarding severe neuropsychiatric effects related to the modulation of dopaminergic pathways, but this was removed in 2017 following the release of the Evaluating Adverse Events in a Global Smoking Cessation Study (EAGLES) [119,120]. In these trials, it was determined that varenicline carries no risk above that of other commonly used nicotine cessation therapies, including NRTs and bupropion [121]. Because of this, along with findings previously discussed, varenicline has been deemed appropriate for monotherapy and is currently recommended as first-line treatment in nicotine cessation by the U.S. Food and Drug Administration [121]. However, the market availability of varenicline has diminished since 2021 following a U.S. Food and Drug Administration recall of Chantix-brand varenicline due to elevated levels of n-nitroso-varenicline [122]. This recall has led to the investigation of alternative pharmaceuticals with similar mechanisms of action to varenicline, such as cytisine.

### 3.7. Emerging Pharmaceutical: Cytisine

While varenicline represents recent advances in smoking cessation pharmacotherapy, its structure and development was based largely on the actions of cytisine, a natural plant alkaloid which has been used in smoking cessation, primarily in Eastern Europe, since the 1960s [123]. Cytisine is a member of the quinolizidine family of alkaloids, sourced from members of the Leguminosae/Fabaceae family, such as *Laburnum*, *Cytisus* and *Lupinus*; the most prominent source for quinolizidine alkaloids like cytisine is the ornamental *Laburnum* tree, which contains an elevated level of alkaloids, especially in its pea-like seeds [124]. Cytisine exerts its effects through the same mechanism as varenicline, which includes competitive partial antagonism of the α4β2 nAChR [125]. While not approved by the U.S. Food and Drug Administration, it is accepted in some markets as an effective, safe, and affordable option for smoking cessation in several countries, including Canada, which labels the drug as a natural health product [126].

In comparative trials, cytisine monotherapy has been shown to improve continuous abstinence at the 1-week, 2-month, and 6-month marks when compared to NRTs [126] and bupropion [127], and no significant difference when compared to varenicline [128]. This, in conjunction with no appreciable adverse effects led to a recent application for approval by the U.S. Food and Drug Administration in 2024, to which the U.S. Food and Drug Administration responded with a request for further data regarding long-term administration, delaying the process by at least one year [129]. Interestingly, in direct comparisons of the efficacy of attaining abstinence, several studies have shown no functional difference in efficacy between cytisine and varenicline [130], which shows a need for future investigation in emergent markets due to the reduction in varenicline prescription given above.

### 3.8. Bupropion, Varenicline, Cytisine Comparison

To better compare the available data on each established or developing pharmaceutical described above, the pharmacokinetic profiles of bupropion, varenicline, and cytisine were compiled into the following Table 3:

Compiling each of the findings above, it is perhaps most interesting that Varenicline had the lowest overall number of contraindications, besides those with impaired renal function and those under 18 years of age [131]. Further, direct comparison studies suggest that varenicline may outperform bupropion in helping former smokers attain nicotine cessation [132]. In the literature, varenicline was observed to have a longer half-life on a less intensive dose (0.5–1.0 mg) with less intensive risk of contravening factors (except an elevated risk of renal toxicity), further corroborating these findings of efficacy.

The developing nature of cytisine as an approved therapeutic meant that there were fewer peer-reviewed or institutional approvals for cytisine compared to the older and more widely approved bupropion and varenicline. Despite this paucity of data, the findings that were available for cytisine showed an effective half-life estimate of 4.8 h off of an initial oral dosage of 1.5–4.5 mg and majority urine excretion profile [133]. A large disparity in reputable pharmacokinetic profiles was observed between cytisine and the other pharmaceuticals described above, suggesting that much more research is needed to widen the understanding of the metabolism and potential contraindications of cytisine use. This places cytisine in a similar class to other emerging pharmaceuticals, which are described below.

### 3.9. Other Pharmaceuticals

Other non-nicotine cessation therapies not approved by the U.S. Food and Drug Administration include nortriptyline, a tricyclic antidepressant (TCA), and clonidine, an alpha-2 adrenergic agonist; however, these agents are considered third-line and only used in cases where typical agents (varenicline, bupropion, NRT) are contraindicated or have failed and psychotherapy has been unsuccessful [89]. These agents, although 2.03 (for nortriptyline) and 1.39–1.63 (for clonidine) times more effective than placebo, respectively [134], are reserved for these rare, treatment resistant cases due to the risk of serious adverse effects [89] which include anticholinergic effects and potential QT prolongation in nortriptyline therapy [89,135,136] and sedation and symptomatic orthostatic hypotension in clonidine therapy [137].

There is also emerging discussion on more experimental therapeutics for smoking cessation, including psilocybin, semaglutide, and essential oils. While initial studies into psilocybin suggest there may be clinical benefit as a tobacco cessation aid [138,139], its continued status as a banned product in Canada, the United States, Latin America, and many European countries precludes its more widespread use. Conversely, the immense market popularity of novel prescription pharmaceuticals like semaglutide and emerging popularity of essential oils have both led to an increase in interest in use applications such as tobacco cessation [140,141,142]. However, these interventions are currently in pre-clinical experimental trials that are still underway as of writing [142,143,144], suggesting that more peer-reviewed data should be collected before definitive prescriptions of GLP-1 agonists and essential oils be recommended as sole therapeutics.

Public and professional opinion on arriving at the ‘best’ modality for nicotine cessation is divided. Nicotine use is exceptionally individualized; each user aligns themselves with a particular set of values, has their own mitigating and motivating factors for continued use, and engages with their own preferred products. Tobacco companies feed on this morass of individuality to sow doubt into the medical profession and keep individual nicotine users engaged with their products [144,145]. The purpose of this assessment was to compile the most recent data and discussion on cessation modalities and assemble each finding into an updated narrative.

## 4. Conclusions

### 4.1. Study Outcomes and Limitations

This study reviewed PubMed, Ovid, Wiley, and Wolters Kluwer to summarize the state of modern tobacco cessation using a clinical, population, and pharmacological approach. In assembling this narrative, this review outlined several sociological, pharmacological, and economic drivers for ongoing nicotine use. In doing so, several research gaps were identified. The first and most pressing need is for the continued assessment of ENDSs and other emerging nicotine products, due to the inconclusive findings on efficacy of ENDS use as cessation aids depicted in Table 2 and Table 3. As ENDS products pre-dominate the modern market, ongoing surveillance will be required to identify and stave off different maladies that will emerge from their use—the nature and pathology of these illnesses have yet to be adequately identified because these products are still so new to the market. Similarly, the search for effective cessation aids will also need be ongoing due to changes in accessibility to prescriptions such as varenicline (as discussed above) and the immense challenge of disentangling ENDSs and other modern nicotine-delivery devices as confounders in the cessation process.

Despite its sprawling assessment, this study does have several limitations. First, the reliance on PubMed, Ovid Wolters Kluwer, and Wiley skewed our search towards English-language, peer-reviewed publications, texts, and clinical trial data available on those hosting networks—this might undercut our findings on some of the available research on emergent products such as cytisine, which has found great folk relevance in several nations where English is not a primary language. There is also the possibility of publication bias due to the reliance of this study on high-impact factor manuscripts hosted by those three sites, which may lead to the sequestration of findings on some products such as cytisine, which has greater a preponderance of use in non-anglophone communities. Further, by its very nature of being a review article, no direct statistical queries could be mined for this analysis, meaning the trend data reported in some parts of the narrative are presented in more general terms, with the reference for the direct study duly included for reader follow-up.

### 4.2. Future Directions

The need for future research findings on emerging pharmaceuticals also mirrors the findings for novel products described above. Our assessment found evidence in support of varenicline being more effective than ENDSs and bupropion in many settings (except in patients who may have renal impairment), but the pharmacokinetics and efficacy of cytisine still require more elucidation. Similarly, the effects of new market drugs like semaglutide, emergent consumer goods like essential oils, and controlled substances like psilocybin need more exploration as well. 

By its very nature, the capital-based world of nicotine addiction and product generation means that each year the dynamic of nicotine consumption will evolve in minor or major stages. In performing this review, we aimed to canvass the current state of research and debate; however, in the next five years the research will shift again. More products will enter the market, be they pharmaceutical cessation aides or novel nicotine-delivery products, and some of the devices, designs, and prescriptions described above will fade into the background. To summarize the findings of this assessment, the standardized clinical hierarchy of cessation tools suggested by the evidence is to begin with approved pharmacotherapies (such as bupropion) as a first-line, reserving NRT for second line therapies, and ENDS products as a distant adjunct. However, modern tobacco control efforts will need to consistently build on existing research and ever-evolve processes of counter-marketing, research, and pharmacological development to stave the tide of nicotine-related illnesses.

## Figures and Tables

**Table 1:** Search queries and total aggregate documents.

Boolean	Sum of Results Retrieved *
“Nicotine Cessation”	2854
“Varenicline”	2769
“Varenicline AND Pharmacokinetics”	98
“E-Cigarettes AND Smoking Cessation”	241
“E-Cigarette Epidemiology”	108
“Cytisine”	1176
“Swedish Snus”	810
“Snus”	5713
“Nicotine AND Gum”	416
“Bupropion”	8949
“Bupropion AND Pharmacokinetics”	572
“Nicotine AND Patches”	782
“Semaglutide AND Cessation”	495
“Psilocybin AND Cessation”	393
“Essential Oil AND Cessation”	24

* Total Records Retrieved: 25,400. Aggregate across all sites.

**Table 2:** Randomized control trials assessing odds of attaining cessation using e-cigarettes.

Author	Year	Country	Population	Cessation Confirmation	Duration of Abstinence	Odds Ratio	95% CI	Notes
Auer et al. [59]	2024	SUI	1246	Serologic and Self-Report	6 Months	1.77	(1.43, 2.20)	
Myers Smith et al. [60]	2022	AUS	135	Serologic and Self-Report	6 Months	6.40	(1.5, 27.3)	ENDS use was compared to NRT alone
Yonek et al. [61]	2021	USA	179	Self-Report Only	7 Days	2.47	(1.2, 5.09)	
Foulds et al. [62]	2022	USA	520	Exhaled CO and Self-Report	7 Days	14	(1.9, 104.9)	Found most significant difference in group that used ENDS product with highest Nicotine content
Bonevski et al. [63]	2025	AUS	363	Self-Report Only	7 Months	1.18	(0.56, 2.54)	
Tattan-Birch et al. [64]	2023	UK	92	Exhaled CO	9–12 Weeks	1.51	(0.91, 2.64)	Assessed ENDS with Varenicline
Klemperer et al. [65]	2025	USA	396	Self-Report	7 Days	1.25	(0.84, 1.85)	
Dawkins et al. [66]	2025	UK	480	Exhaled CO and Self Report	1–24 Weeks	2.43	(0.51, 11.64)	
Smith et al. [67]	2025	USA	30	Exhaled CO and Self Report	7 Days	4.8	(0.5, 46.5)	

Abbreviation Guide: 95% CI: Confidence Interval, SUI: Switzerland, AUS: Australia, USA; United States of America, UK: United Kingdom, CO: Carbon Monoxide.

**Table 3:** Compiled pharmacokinetic profiles for bupropion, varenicline, and cytisine.

Drug	Dosage (mg)	Half Life (Hours)	Range (Hours)	Metabolism	Elimination	Contraindications
Bupropion	100–250	14	8–14	CYP2B6 Isozyme	87% Urine, 10% Fecal	Liver Disease, CHF
Varenicline	0.5–1.0	24	3–24+	OCT2	92% Urine	Renal Impairment, Pediatrics
Cytisine	1.5–4.5	4.8	1–24+	Excreted as Unchanged Drug [131]	Majority Urine	Developing

Where: CHF: Congestive Heart Failure, OCT2: Organic Cation Transporter, +: unspecified duration beyond 24 h.

## Data Availability

No new data were created or analyzed in this study. Data sharing is not applicable to this article.

## Abbreviations

The following abbreviations are used in this manuscript:

ENDS	Electronic Nicotine-Delivery System
MAOI	Monoamine Oxidase Inhibitor
TCA	Tricyclic Antidepressant
DA	Dopamine
NE	Norepinephrine
NRT	Nicotine Replacement Therapy
NACHR	Nicotine Acetylcholine Receptor
SSRI	Selective Serotonin Reuptake Inhibitor
AUS	Australia
USA	United States of America
SUI	Switzerland
UK	United Kingdom
CO	Carbon Monoxide
CHF	Congestive Heart Failure
OCT2	Organic Cation Transporter
CI	Confidence Interval
